# A Dose Too Far: Valacyclovir’s Surprising Side Effect

**DOI:** 10.7759/cureus.84500

**Published:** 2025-05-20

**Authors:** Sofia Cestero, Trevor Wolchover, Harpreet S Dosanjh, Saro Avedikian, Jasprit Takher

**Affiliations:** 1 Internal Medicine, Los Robles Regional Medical Center, Thousand Oaks, USA; 2 Internal Medicine, Los Robles Regional Medical Center, Westlake Village, USA

**Keywords:** end stage renal disease (esrd), herpes simplex, pharma, valcyclovir, valtex

## Abstract

Valacyclovir, a prodrug of acyclovir, is a widely prescribed antiviral medication known for its efficacy in treating herpes infections. Although generally considered safe, rare cases of psychiatric adverse effects, including psychosis, have been reported. The precise mechanism underlying valacyclovir-induced psychosis remains unclear, but proposed explanations include potential neurotoxicity and alterations in neurotransmitter activity. Importantly, such adverse effects have been documented even in patients with normal renal function. Given that valacyclovir's pharmacokinetics are significantly altered in individuals with renal impairment - characterized by reduced clearance and prolonged half-life - the risk of drug accumulation and subsequent neurological complications is heightened in this population. Here, we present a unique case of valacyclovir-induced psychosis in a patient with pre-existing renal failure, emphasizing the importance of considering renal function when prescribing and monitoring this medication to mitigate the risk of psychiatric complications.

## Introduction

Valacyclovir, a prodrug of acyclovir, is a widely prescribed antiviral medication known for its efficacy in treating herpes infections. Although generally considered safe, rare cases of psychiatric adverse effects, including psychosis, have been reported. The precise mechanism underlying valacyclovir-induced psychosis remains unclear, but proposed explanations include potential neurotoxicity and alterations in neurotransmitter activity. Importantly, such adverse effects have been documented even in patients with normal renal function [1,2]. Given that valacyclovir's pharmacokinetics are significantly altered in individuals with renal impairment - characterized by reduced clearance and prolonged half-life - the risk of drug accumulation and subsequent neurological complications is heightened in this population [2,3]. Here, we present a unique case of valacyclovir-induced psychosis in a patient with pre-existing renal failure, emphasizing the importance of considering renal function when prescribing and monitoring this medication to mitigate the risk of psychiatric complications.

## Case presentation

In this report we present a 53-year-old man with a medical history significant for hypertension, insulin-dependent diabetes mellitus and end-stage renal disease (ESRD) on daily peritoneal dialysis who was brought to the emergency department in October of 2023 by his wife due to increasing agitation and confusion. About five days prior to presenting at the hospital, the patient began experiencing changes in his behavior that worsened as time went on. Initially, he only experienced headaches and mild confusion, including forgetting things that were mentioned to him just minutes prior. These symptoms progressed to increased paranoia and agitation. The patient’s son reported that visual and auditory hallucinations were involved preceding hospitalization, during which the patient was found to be responding to internal stimuli several times throughout the day. He would engage in conversations with people who were not present. The hallucinations and cognitive impairment progressively worsened to the point that the patient developed nonsensical speech and experienced slurring of his speech. His family denied any previous psychiatric history.

Prior to these psychological symptoms, the patient had no changes in his medication regimen or dialysis treatments other than a new-onset rash on his right shoulder. Just one week prior to presenting at the hospital, three days prior to psychological symptoms, the patient was seen at an Urgent Care for a new-onset painful rash. Subsequently the rash was determined to be non-disseminated shingles and the patient was started on oral valacyclovir, with a dose of 1000 mg every eight hours. After two days of taking valacyclovir, his psychological symptoms began.

**Figure 1:**
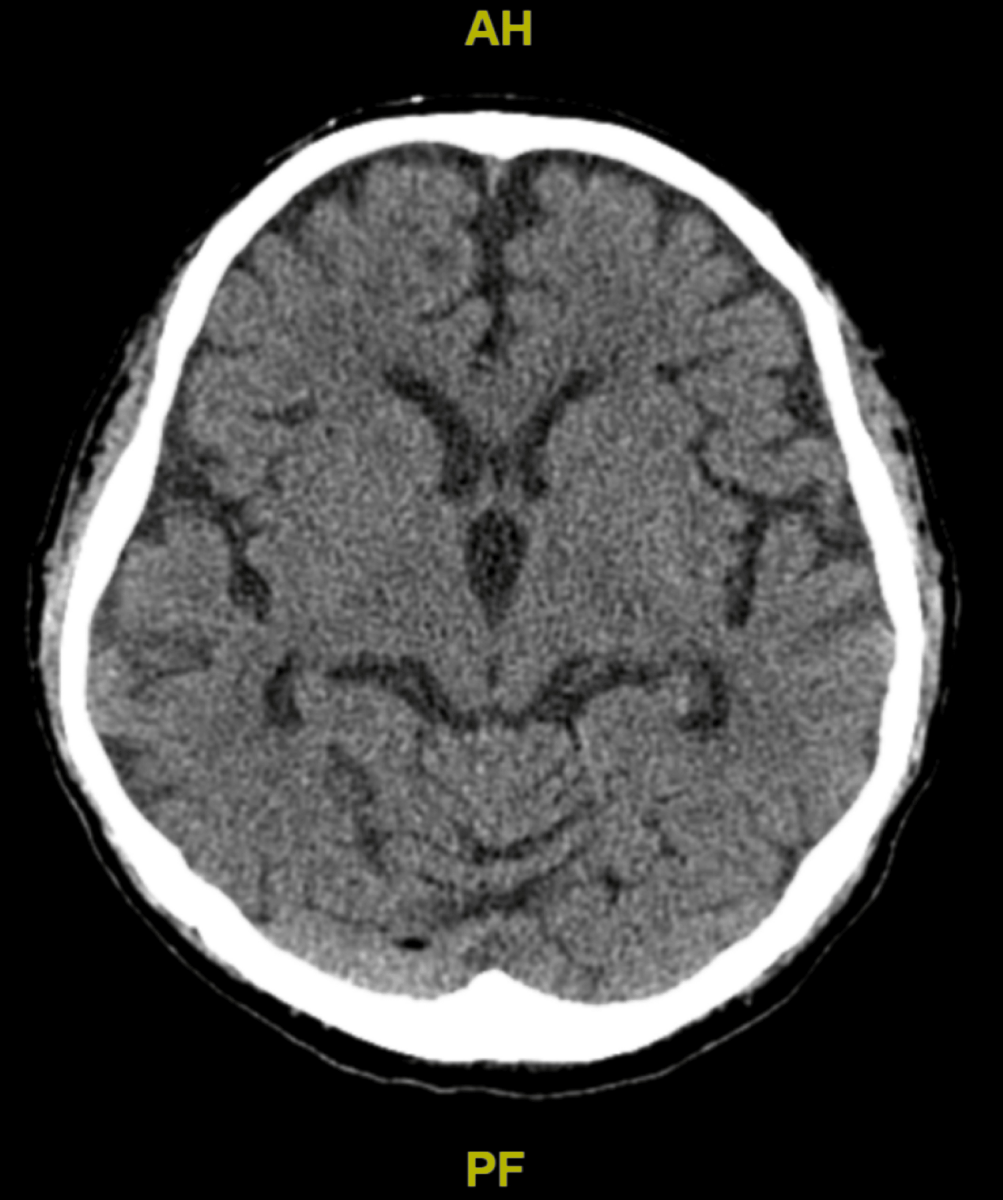
CT head without contrast. Negative for any acute intracranial pathology including signs of inflammation, stroke, hemorrhage, masses or midline shift.

On admission, the patient was intubated, as his increasing agitation and confusion raised concern for airway compromise. Encephalitis was suspected as the culprit for his acute changes in mental status and recent shingles infection. He was started on vancomycin, piperacillin/tazobactam and acyclovir, all of which were renally dosed, to cover for potential causes of infection. At the time of admission, a CT head was obtained to rule out possible stroke, imaging was negative for signs of inflammation or stroke/hemorrhage (Figure 1). The patient's CT head was unremarkable, as shown in Figure 1. A lumbar puncture was performed during this time, which resulted in no signs of inflammation or infection. The presence of herpes simplex virus (HSV), varicella-zoster virus (VZV), West Nile virus and gram stains/cultures were negative in the lumbar puncture. 

Due to negative infectious workup, the cause of the patient’s psychological symptoms was determined to be caused due to valcyclovir neurotoxicity. He was restarted on daily peritoneal dialysis and all antibiotics and anti-viral medications were discontinued. The patient was able to be successfully extubated and his cognitive function returned to baseline on discharge.

## Discussion

This case is notable due to the relatively rare presentation of valacyclovir-induced psychosis in a patient with ESRD. While neurotoxicity associated with acyclovir has been more extensively documented, particularly in the context of renal impairment, reports involving valacyclovir are comparatively sparse, contributing to under-recognition of this complication in clinical practice [1,2]. In this patient, it was established that valcyclovir was the culprit for his ongoing psychiatric symptoms. 

Although valacyclovir is a prodrug of acyclovir and shares a similar antiviral mechanism of action, its pharmacokinetic profile differs slightly in regards to its metabolites, especially in patients with compromised renal function. Valacyclovir is rapidly converted to acyclovir in the liver and intestines, with subsequent renal excretion. In patients with ESRD, impaired clearance leads to accumulation of the drug and its active metabolites, prolonging the half-life and increasing the risk of neurotoxic effects [2,3]. Both medications have been associated with neurotoxic effects, which include confusion, hallucinations, agitation and psychosis. 

A population-based study demonstrated that the incidence of altered mental status in ESRD patients undergoing dialysis was 1.68 per 1,000 person-days, highlighting the vulnerability of this population to central nervous system (CNS) complications related to antiviral therapy [3]. Neurotoxicity associated with elevated serum acyclovir levels can manifest as a spectrum of neuropsychiatric symptoms, including confusion, agitation, hallucinations, and overt psychosis [1,4]. These effects are believed to stem from acyclovir’s potential to cross the blood-brain barrier and disrupt neurotransmitter function, possibly through excitatory pathways or mitochondrial dysfunction.

Case literature further supports the occurrence of valacyclovir-induced neurotoxicity, with reports ranging from delirium and visual hallucinations to more severe psychiatric syndromes such as pseudobulbar affect, particularly in patients on peritoneal dialysis due to ESRD [4-6]. For instance, Memon et al. described a patient on peritoneal dialysis who developed pseudobulbar symptoms and altered mental status, which resolved after cessation of valacyclovir and intensification of the patient's dialysis regimen [5]. Similarly, Kassam and Cunningham reported a striking case of Cotard syndrome, which is characterized by nihilistic delusions, resulting from valacyclovir toxicity in a renally impaired patient [6-8]. The patient in this report experienced similar side effects, as documented in previous case reports. He experienced altered mentation, hallucinations that included auditory and visual components, but more importantly, his symptoms emerged when taking valcyclovir. 

Given these findings, it is essential for clinicians to remain vigilant when prescribing valacyclovir or acyclovir in patients with renal dysfunction. Dose adjustments based on estimated glomerular filtration rate (eGFR) and close monitoring for neuropsychiatric symptoms are critical. Prompt recognition and discontinuation of the offending agent can lead to rapid and complete resolution of symptoms, as demonstrated in our patient. It has also been found that patients experiencing neurological side effects can benefit from intensification of their dialysis regimen in order to alleviate the effects of the medication [7,8]. This case reinforces the importance of individualized dosing and heightened awareness of potential neurotoxicity, even with commonly used antiviral agents. 

## Conclusions

In summary, we presented a case of a patient with ESRD who was treated for shingles with the use of Valtrex in the outpatient setting. The cause of his altered mentation was determined to be Valtrex as the patient’s status significantly improved following several rounds of dialysis and cessation of the medication. This case emphasizes the critical need for careful monitoring and dose adjustments when prescribing valacyclovir or acyclovir in patients with ESRD or renal impairment. Regular assessment of renal function, adherence to recommended dosing guidelines, and prompt recognition of neurotoxic symptoms are essential to minimizing the risk of psychiatric complications. By incorporating these precautions into clinical practice, healthcare providers can enhance patient safety and improve outcomes in this vulnerable population.
